# Digital development: a database of cell lineage differentiation in *C. elegans* with lineage phenotypes, cell-specific gene functions and a multiscale model

**DOI:** 10.1093/nar/gkv1119

**Published:** 2015-10-25

**Authors:** Anthony Santella, Ismar Kovacevic, Laura A. Herndon, David H. Hall, Zhuo Du, Zhirong Bao

**Affiliations:** 1 Sloan Kettering Institute, New York, NY 10065, USA; 2 Albert Einstein College of Medicine, Bronx, NY 10461, USA; 3 Institute of Genetics and Developmental Biology, Chinese Academy of Sciences, Beijing 100101, China

## Abstract

Developmental systems biology is poised to exploit large-scale data from two approaches: genomics and live imaging. The combination of the two offers the opportunity to map gene functions and gene networks *in vivo* at single-cell resolution using cell tracking and quantification of cellular phenotypes. Here we present Digital Development (http://www.digital-development.org), a database of cell lineage differentiation with curated phenotypes, cell-specific gene functions and a multiscale model. The database stores data from recent systematic studies of cell lineage differentiation in the *C. elegans* embryo containing ∼200 conserved genes, 1400 perturbed cell lineages and 600 000 digitized single cells. Users can conveniently browse, search and download four categories of phenotypic and functional information from an intuitive web interface. This information includes lineage differentiation phenotypes, cell-specific gene functions, differentiation landscapes and fate choices, and a multiscale model of lineage differentiation. Digital Development provides a comprehensive, curated, multidimensional database for developmental biology. The scale, resolution and richness of biological information presented here facilitate exploration of gene-specific and systems-level mechanisms of lineage differentiation in Metazoans.

## INTRODUCTION

Phenomic data characterizing Metazoan development are valuable resources for understanding *in vivo* gene function, genetic networks and developmental mechanisms. Recent advances in live imaging and image bioinformatics offer the opportunity to dissect complex developmental processes at high spatiotemporal resolutions. Many *in vivo* developmental processes can be recorded and analyzed at single-cell resolution using 3D time-lapse imaging followed by systematic cell tracking and quantification of developmental phenotypes for individual cells (1–3). The nematode *Caenorhabditis elegans* is a leading model organism for systematic single-cell analysis of Metazoan development (4). Due to its transparent cuticle and invariant cell lineage (5), every *C. elegans* cell can be traced, analyzed and compared between genotypes on a cell-by-cell basis. The combination of systems-wide perturbations and live-imaging-based phenotypic analysis is poised to generate large-scale cellular phenomic data that will allow dissection of *in vivo* gene functions and network operation in single cells to guide and drive development.

Cell lineage differentiation is an important step of development during which the totipotent zygote differentiates into specialized cell types. Mapping and analysis of lineage differentiation phenotypes is critical for a mechanistic understanding of *in vivo* cell fate differentiation and *in vitro* cell fate engineering. However, perturbation-based experimental data are scarce, especially those with manually curated, high quality results in terms of cell tracking and differentiation phenotypes. Recently, we have conducted systematic analyses of lineage differentiation in the *C. elegans* embryo (6–8). We perturbed 204 conserved regulatory genes by RNAi and used 3D time-lapse imaging to record the first half of embryogenesis. Through direct lineage tracing and quantifying the expression of multiple tissue-specific markers we have generated a comprehensive phenomic dataset with 1368 individual embryos and approximately 593 000 digitized cells. These data were manually curated and are of high quality. Phenotypic analyses further revealed cell-specific gene functions, differentiation trajectories and a multiscale model of lineage differentiation.

To allow users from diverse fields to access and explore this rich dataset, we created a comprehensive database named Digital Development (http://www.digital-development.org) to provide a platform to browse, search and download the raw and processed data from phenotypic and functional studies. We envision that Digital Development will complement the existing databases that document *C. elegans* biology. Notably, WormBase (9) serves as a genome database where comprehensive biological information is organized. WormAtlas (http://www.wormatlas.org) serves as a knowledge base that features behavioral and structural anatomy, with extensive collections of images and annotation. Several databases are also available that are focused on live-imaging and lineaging in the early embryo, with EPIC focused on cellular gene expression patterns (10), WDDD (11) on cell position dynamics, as well as RNAiDB (12), PhenoBank (13,14) and Phenics (15) on cell cycle regulation. In comparison, Digital Development is focused on cell fate decisions and cell lineage differentiation. As noted below, to maximize its value to users, Digital Development is systematically linked to WormBase and WormAtlas. The information on cellular differentiation phenotypes, *in vivo* gene functions and systems-level models of fate regulation in Digital Development will be a valuable new resource for exploring the regulation of lineage differentiation not only in *C. elegans* but also in Metazoan development in general.

## THE UNDERLYING BIOLOGICAL INFORMATION

### Gene information

The database includes phenotypic data for 204 conserved essential genes. These genes induce embryonic lethality (Emb) phenotypes when knocked down, are conserved in humans, and play regulatory roles for broadly interesting molecular and cellular processes.

### Phenotypic information

**Figure 1.**
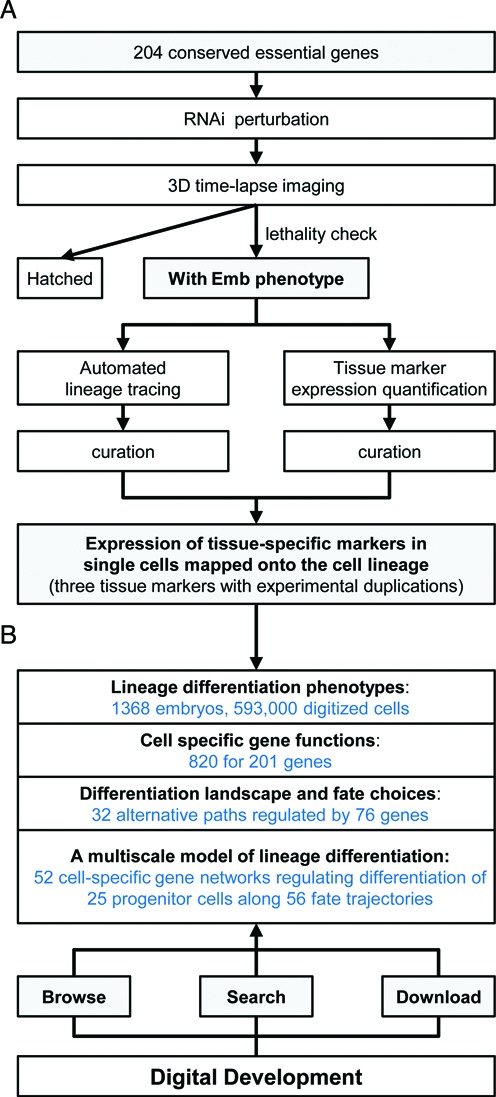
Overview of Digital Development database. (**A**) Flow chart shows key steps used to assay lineage differentiation phenotypes. For details see the main text. (**B**) Key information that can be browsed, searched and downloaded in the database.

An integrated pipeline was applied to perform high-dimensional phenotypic analysis of lineage differentiation over space, time and genome (Figure 1). We used RNAi to knock down each gene and 3D time-lapse imaging to record the first 6–8 h of embryogenesis (3,6,16). Effectiveness of RNAi was systematically monitored so that only image series containing embryos with the Emb phenotype were further processed. Lineage differentiation phenotypes were assayed by direct lineage tracing (based on ubiquitously expressed mCherry) and quantification of tissue-specific marker expression (based on GFP-tagged tissue-specific proteins) in individual cells. Lineage tracing was performed automatically using StarryNite (16–18) followed by multiple rounds of systematic manual curation. The entire lineage of most perturbed embryos was traced through the first 9 rounds of cell divisions, or about 350 cells. The estimated cumulative accuracy of cell tracking post-curation is 98% (8). Three tissue-specific reporters with highly reproducible cellular expression patterns were used to profile differentiation of four types of major tissues: pha-4 for pharynx and gut (19), cnd-1 for a subset of motor neurons (20) and nhr-25 for major hypodermis cells (21,22). These markers cover 61% of the cell lineage and all three germ layers. Lineage differentiation phenotypes were digitized as cellular expression of tissue markers mapped onto the cell lineage. For each gene, the phenotypes were assayed in multiple embryos for three tissue markers and several experimental replicates. Wild type expression was characterized using the same methods over 10 embryos per marker, results are highly consistent. The consensus wild-type expression pattern for each marker is used in the database for visualizations and comparisons. In total, the database includes 1368 perturbed embryonic lineages containing roughly 593 000 digitized cells with quantitative measurements of fate marker expression.

### Functional information

The raw phenotypic data were further processed in a variety of ways to analyze cell-specific gene functions and systems-level regulation of lineage differentiation. For gene functions, raw data were processed to identify phenotypic changes for individual embryos and genes, and to infer primary phenotypes and function sites for each gene. Many of the phenotypes and functions have been verified with additional experiments (6–8). For understanding systems-level regulation of lineage differentiation, data were processed to identify the regulator of binary fate choices, infer trajectories of cell differentiation and construct a multiscale model of lineage differentiation. Detailed information on data processing is described elsewhere (6,8).

## DATABASE CONTENT AND STRUCTURE

We organized and cross referenced phenotypic and functional information to allow users to conveniently browse, search and download four categories of information: (i) lineage differentiation phenotypes, (ii) cell-specific gene functions, (iii) differentiation trajectories and fate choices and (iv) a multiscale model of lineage differentiation.

### Lineage differentiation phenotypes

**Figure 2.**
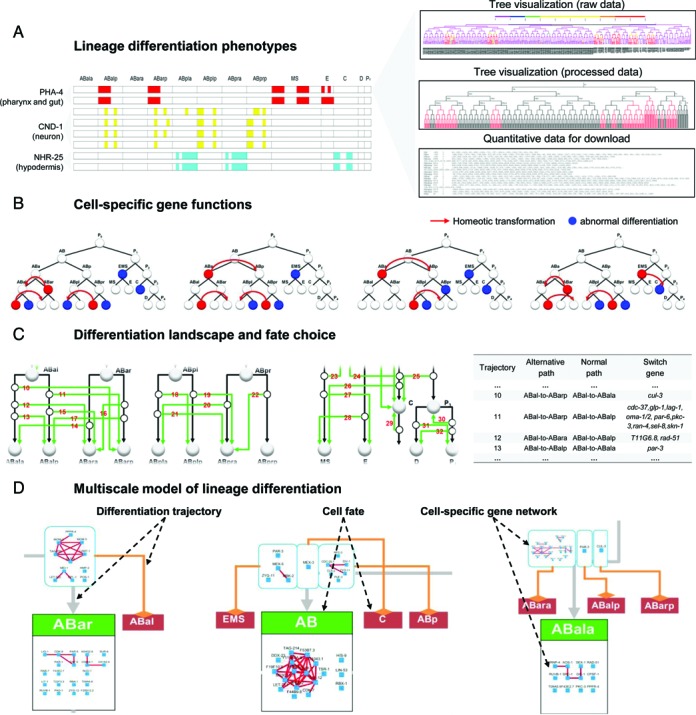
Example of digital development. (**A**) Lineage differentiation phenotypes. Phenotypes are organized first by genes and then by tissue-markers and finally by experimental replications. Phenotypes are provided in multiple forms for convenience in comparing and visualizing phenotypes. First, a heatmap summary of marker expression patterns for individual embryos (one embryo per row) is provided for each gene (left panel). Second, color-coded representation of tissue marker expression for each embryo is provided for detailed analysis of phenotypes (top, right panel). Vertical lines represent cells and horizontal lines represent cell divisions. Expression level is shown in a color gradient. Third, a standardized representation of phenotypes is provided where the cell lineage topology is standardized and expression status is binarized for the ease of comparison (middle, right panel). Finally, the quantitative data of tissue marker expression in each cell at each time point is available for download (bottom, right panel). (**B**) Cell-specific gene functions. Figure shows examples in which gene action sites and functions are graphically summarized. In each panel, the standard cell lineage that leads to the 13 founder cells is represented as white nodes (cells) connected by black lines (mother–daughter relationship). Gene functions are mapped onto specific cells: red nodes represent cases of homeotic transformations with arrows representing the type and direction of fate transformation; blue nodes represent cases where the lineage differentiation in the sublineage of a given progenitor cell is affected by gene perturbation. (**C**) Differentiation trajectories and fate choices. Left panel: nodes represent cell fates and arrows represent trajectories of differentiation. Black arrows represent trajectories observed during normal development; green arrows represent alternative trajectories observed due to gene perturbation. All detected alternative trajectories are labeled by number. Each open circle represents a decision point of cell fate choice. Right panel: regulation of the fate choice between default and alternative trajectories. (**D**) A multiscale model of lineage differentiation. Figure shows part of the model in which cell fates (rectangular boxes, below) are linked by differentiation trajectories (arrows) and regulated by gene networks (cyan nodes linked by red edges). Black arrows pointing to green boxes represent the trajectories that lead to the wild-type cell fates and orange arrows pointing to dark red boxes represent alternative differentiation trajectories. Genes are organized in two types of cell-specific networks. Those that regulate cell fate choice (cyan boxes) are placed on the trajectories that lead to the alternative fate; those that regulate fate differentiation are placed in the boxes corresponding to the original fates.

The database includes the phenotypic data of 1368 embryos for 204 gene perturbations. Phenotypes are represented by tissue-marker expression in single cells mapped onto the cell lineage of each perturbed embryo and are organized by genes (Figure 2A). The database allows users to explore the phenomic data in various ways. First, a summary heatmap of the marker expression pattern across the lineage is provided for each embryo. Marker expression in the terminal cell (350-cell stage) is used is to show clonal expression of tissue markers. Cells are ordered based on cell lineage and the founder cell names are listed on top. Different markers are in different colors and each block corresponds to a uniform and significant clone (sublineage) that expresses a tissue marker. For each gene, the heatmaps are compiled across multiple tissue markers and experiments (Figure 2A, left panel). This provides a quick view of the phenotypes for a gene of interest. Second, detailed phenotypes for each embryo are also provided for in-depth analysis (Figure 2A, right panel). For each embryo, quantitative marker expression in individual cells at each time point (75 s interval) is visualized as a color-coded tree and the source data is available for download. Each tree shows the cell lineage and the tree branch color shows tissue marker expression levels. Two types of visualization are provided. One shows the raw phenotype detection in which the tree topology is based on direct lineage tracing and the color gradient is proportional to marker expression level (10,23). The other shows the processed phenotypic detection results in which the tree topology is standardized and the expression status (on/off) is binarized for ease of comparison (6). Lineage differentiation phenotypes for specific genes and embryos can be accessed using the browse and search functions.

### Cell specific gene functions

The cellular phenotypes were processed to infer primary phenotypic changes (6). The database includes 820 predicted primary cell fate changes, each indicating a cell-specific regulatory function for a gene. The syntax of gene functional annotation is ‘a gene regulates these aspects of lineage differentiation in this specific progenitor cell’. Two types of regulatory role were identified: those that induced homeotic transformations (175 instances) are considered regulatory switches of binary cell fate choice; those that induced other abnormalities in lineage differentiation are considered as regulators of cell fate differentiation. The database provides an intuitive graphic representation (Figure 2B) of cell-specific gene functions, which are available for browsing, search and batch download.

### Differentiation landscape and fate choices

All inferred binary fate choices were integrated with normal fate progression to generate a landscape of lineage differentiation with 56 trajectories and 32 types of regulated fate choices. The graph (Figure 2C) allows users to explore all possible trajectories a cell can take to differentiate into a specific fate and to find the corresponding regulatory switch genes that determine the differentiation path along the trajectories.

### A multiscale model of lineage differentiation

A gene regulatory network is constructed based on phenotypic similarity between genes and integrated with the individual cells in the differentiation landscape to generate a multiscale model (gene→cell→landscape) of lineage differentiation. The model contains 25 progenitor cell fates, 56 fate trajectories, and 52 cell-specific gene networks. The database provides multiple ways to explore the model. First, the high-resolution graph and source data of the model are available for download. Second, it allows the user to search cell-specific networks that regulate fate choice and differentiation of individual progenitor cells (search by cell). Third, it allows the user to search networks that regulate all possible differentiation trajectories leading to a specific cell fate (search by fate).

## FUTURE DEVELOPMENT

In addition to lineage differentiation, many other developmental phenotypes can readily be extracted from images to investigate the regulatory mechanisms of proliferation and morphogenesis. We plan to process and deposit the cell cycle and position phenotypes for each cell in each perturbed embryo. We also plan to share the original 4D image series for researchers to reanalyze the phenotypes and extract additional phenotypes from this experimental dataset. Since developmental phenotypes are inherently complex and not intuitive to describe in static formats, we plan to enhance the capacity for interactive phenotype visualization. To facilitate interactive and visual exploration of the data in a spatiotemporal manner, we plan to link the data to WormGUIDES (24), a recently developed interactive interface for exploring cellular behaviors of development in 4D.

## CONCLUSION

Digital Development (http://www.digital-development.org) provides a comprehensive, curated, multidimensional database for developmental phenotypes, cell-specific gene functions, differentiation trajectories and a multiscale model of lineage differentiation in the *C. elegans* embryo. The database is also available through WormAtlas (http://www.wormatlas.org). Digital Development will be a valuable resource and venue for exploring *in vivo* gene function, gene regulatory networks, mechanisms of cell differentiation and systems-level properties of development.
